# Leptin produced by obese adipose stromal/stem cells enhances proliferation and metastasis of estrogen receptor positive breast cancers

**DOI:** 10.1186/s13058-015-0622-z

**Published:** 2015-08-19

**Authors:** Amy L. Strong, Jason F. Ohlstein, Brandi A. Biagas, Lyndsay V. Rhodes, Dorothy T. Pei, H. Alan Tucker, Claire Llamas, Annie C. Bowles, Maria F. Dutreil, Shijia Zhang, Jeffrey M. Gimble, Matthew E. Burow, Bruce A. Bunnell

**Affiliations:** Center for Stem Cell Research and Regenerative Medicine, Tulane University School of Medicine, 1430 Tulane Avenue, SL-99, New Orleans, LA 70112 USA; Department of Medicine, Section of Hematology and Medical Oncology, Tulane University Health Sciences Center, New Orleans, LA 70112 USA; Departments of Medicine and Surgery, Tulane University Health Sciences Center, New Orleans, LA 70112 USA; Department of Pharmacology, Tulane University School of Medicine, New Orleans, LA 70112 USA

## Abstract

**Introduction:**

The steady increase in the incidence of obesity among adults has been paralleled with higher levels of obesity-associated breast cancer. While recent studies have suggested that adipose stromal/stem cells (ASCs) isolated from obese women enhance tumorigenicity, the mechanism(s) by which this occurs remains undefined. Evidence suggests that increased adiposity results in increased leptin secretion from adipose tissue, which has been shown to increased cancer cell proliferation. Previously, our group demonstrated that ASCs isolated from obese women (obASCs) also express higher levels of leptin relative to ASCs isolated from lean women (lnASCs) and that this obASC-derived leptin may account for enhanced breast cancer cell growth. The current study investigates the impact of inhibiting leptin expression in lnASCs and obASCs on breast cancer cell (BCC) growth and progression.

**Methods:**

Estrogen receptor positive (ER+) BCCs were co-cultured with leptin shRNA lnASCs or leptin shRNA obASCs and changes in the proliferation, migration, invasion, and gene expression of BCCs were investigated. To assess the direct impact of leptin inhibition in obASCs on BCC proliferation, MCF7 cells were injected alone or mixed with control shRNA obASCs or leptin shRNA obASCs into SCID/beige mice.

**Results:**

ER+ BCCs were responsive to obASCs during direct co-culture, whereas lnASCs were unable to increase ER^+^ BCC growth. shRNA silencing of leptin in obASCs negated the enhanced proliferative effects of obASC on BCCs following direct co-culture. BCCs co-cultured with obASCs demonstrated enhanced expression of epithelial-to-mesenchymal transition (EMT) and metastasis genes (SERPINE1, MMP-2, and IL-6), while BCCs co-cultured with leptin shRNA obASCs did not display similar levels of gene induction. Knockdown of leptin significantly reduced tumor volume and decreased the number of metastatic lesions to the lung and liver. These results correlated with reduced expression of both SERPINE1 and MMP-2 in tumors formed with MCF7 cells mixed with leptin shRNA obASCs, when compared to tumors formed with MCF7 cells mixed with control shRNA obASCs.

**Conclusion:**

This study provides mechanistic insight as to how obesity enhances the proliferation and metastasis of breast cancer cells; specifically, obASC-derived leptin contributes to the aggressiveness of breast cancer in obese women.

**Electronic supplementary material:**

The online version of this article (doi:10.1186/s13058-015-0622-z) contains supplementary material, which is available to authorized users.

## Introduction

Obesity is defined by the accumulation of excessive adipose tissue that can contribute to physical and psychosocial impairment. The prevalence of obesity in the world, particularly in the USA, has increased over the past four decades, with one third of adults in the USA meeting the criteria for obesity [1]. As a result, there has been an increase in the incidence of obesity-associated cancers [2–4]. More specifically, recent studies suggest that obesity increases the incidence of breast cancer [5, 6].

Epidemiological studies investigating the role of obesity in breast cancer suggest that obesity increases the incidence of metastatic breast tumors, results in higher rates of incidence of recurrence, and increases mortality. Haakinson et al. found that obese patients are diagnosed with larger primary tumors and had increased incidence of lymph node metastases [7]. Furthermore, in postmenopausal breast cancer patients, up to 50 % of deaths have been attributed to obesity [8]. While the link between obesity and breast cancer has been well-documented from epidemiologic analyses, the molecular mechanisms underlying this correlation are not fully defined.

An analysis of the interplay between breast cancer and obesity provides some insights into the underlying pathophysiology. During breast cancer development and progression, a complex multi-step cascade converts normal breast epithelial cells into malignant cells [9–11]. One of the key steps involves the interaction between the epithelial cells and the stromal microenvironment, which contains adipose stromal/stem cells (ASCs) [12]. Studies have shown that obesity significantly increases the number of ASCs within the adipose tissue. This ASC hyperplasia has been shown to support both angiogenesis and adipogenesis and to alter the gene expression profile of ASCs such that they enhance cancer growth [13–15]. Recently, our group has demonstrated that ASCs isolated from obese patients with body mass index (BMI) ≥30 (obASCs) enhance the tumorigenicity MCF7 breast cancer cells, and alter their gene expression profile [13]. Additionally, the data showed that the obASCs expressed significantly higher levels of leptin compared to ASCs isolated from lean patients with BMI ≤25 (lnASCs). However, the overexpression of leptin in obASCs and the impact it has on increasing the aggressiveness of tumor cell biology in vitro and in vivo has not been investigated.

The role of leptin produced by obASCs on breast cancer cells (BCCs) was investigated in this study by inhibiting the expression of leptin using a short hairpin RNA (shRNA) knockdown strategy. The obASCs preferentially increased the proliferation, migration, and invasion of several estrogen receptor positive (ER^+^) BCC lines, including MCF7, ZR75, and T47D, during direct co-culture. Reducing the levels of leptin in obASCs negated their effects on BCCs. Consistent with phenotypic changes, inhibiting leptin expression in obASCs negated alterations to the gene expression profile of BCC after co-culture. Furthermore, reducing leptin levels in obASCs also resulted in a reduction in tumor volume and fewer metastatic lesions in the lung and liver of SCID/beige mice. These results implicate obASC-derived leptin as a key mechanism that alters BCC growth and supports changes to the biology of BCCs into a more aggressive phenotype. The results here suggest that the inhibition of leptin secreted by obASCs may result in reduced tumor volume and metastasis to distant organs, reducing the burden of obesity-associated breast cancers.

## Methods

### Human subjects

Human ASCs were obtained from 12 Caucasian women (two groups, six donors per group) undergoing elective liposuction procedures, as previously described [16]. All protocols were reviewed and approved by the Pennington Biomedical Research Center Institutional Review Board, and all human participants provided written informed consent. Briefly, ASCs were isolated from processed liposuction aspirates from the subcutaneous abdominal adipose tissue of lean or obese patients. Liposuction aspirates were incubated in 0.1 % type I collagenase (Sigma , St. Louis, MO) and 1 % powdered bovine serum albumin (BSA, fraction V; Sigma) dissolved in 100 ml of PBS supplemented with 2 mM calcium chloride. The mixture was placed in a 37 °C shaking water-bath or incubator at 75 rpm for 60 minutes and then centrifuged to remove oil, fat, primary adipocytes, collagenase solution and cellular debris. The resulting cell pellet was re-suspended in stromal medium (SM), which consisted of DMEM/F12 (Hyclone, Logan, UT, USA), 10 % FBS (Hyclone), 1 % antibiotic/antimycotic (Fisher Scientific, Houston, TX, USA), and plated in 175-cm^2^ flasks. Fresh SM was added every 2–3 days until cells achieved 80–90 % confluence, at which time cells were harvested with 0.25 % trypsin/1 mM EDTA (GIBCO, Grand Island, NY, USA) and cryopreserved prior to experimental use. The mean BMI for the lnASC group was 22.7 ± 1.9, while the mean BMI for the obASCs was 32.7 ± 3.7. The mean age of the subjects for each group of donors was as follows: lnASCs (38.8 ± 7.0 years) and obASCs (42.5 ± 8.9 years). There was no statistically significant difference in age between the donor groups.

### Cell culture

#### ASCs

Frozen vials of ASCs were thawed and cultured on 150-cm^2^ culture dishes (Nunc, Rochester, NY, USA) in 25 ml of complete culture medium (CCM) and incubated at 37 °C with 5 % humidified CO_2_. After 24 hours, viable cells were harvested with 0.25 % trypsin/1 mM EDTA and re-plated at 100–200 cells/cm^2^ in CCM, which consisted of α-Minimal Essential Medium (αMEM; GIBCO), 20 % FBS (Atlanta Biologicals, Lawrenceville, GA, USA), 100 units per ml penicillin/100 μg/ml streptomycin (P/S; GIBCO), and 2 mM L-glutamine (GIBCO). Medium was changed every 2–3 days. For all experiments, sub-confluent cells (≤70 % confluent) between passages 2–6 were used. Characterization of stem cells has previously been performed and published [13].

#### Breast cancer cell (BCC) lines

MCF7 (HTB-22), ZR75 (CRL-1500), and T47D (HTB-133) cells were obtained directly from American Type Culture Collection (ATCC; Manassas, VA, USA) and used within 6 months of resuscitation. Cell line authentication was conducted by ATCC via short tandem repeat profiling. Cells were cultured in DMEM (GIBCO), supplemented with 10 % FBS (Atlanta Biologicals) and P/S. Cells were grown at 37 °C with 5 % humidified CO_2_, fed every 2–3 days, and split 1:4 to 1:6 when the cells reached 90 % confluence.

### Synthesis of green fluorescent protein positive (GFP^+^) BCCs

To produce lentivirus, 293T cells were transfected through a modified calcium chloride transfection protocol when cells reached 70−75 % confluence. For each transfection, 10 μg of packaging plasmid, enveloping encoding plasmid, and transfer plasmid containing GFP and neomycin resistance or dsRed and neomycin resistance were used. After 48 hours, medium was harvested and used to transduce cancer cells. To transduce ASCs, conditioned medium containing virus with dsRed and neomycin resistance was added to ASCs at 70 % confluence. MCF7, ZR75, T47D, and ASCs were selected with 500 μg/ml of Geneticin (Invitrogen; Carlsbad, CA, USA) for 2 weeks and GFP expression or dsRed expression was verified with flow cytometry. All cancer cells and ASCs used for experimentation expressed GFP or dsRed unless otherwise specified.

### Conditioned media

ASCs pooled from six donors per group, were plated on a 150-cm^2^ cell culture dish at 100 cells/cm^2^. After overnight culture, medium was replaced with serum-free αMEM. After 7 days, conditioned media (CM) were collected and filtered to remove cellular debris. The total number of ASCs was also counted to verify equal number of cells after 7 days. ASC CM from lnASCs and obASCs was plated on top of BCCs set up in triplicates. After 7 days, the total number of MCF7 cells was counted. Where indicated, CM was collected from control shRNA lnASCs, leptin shRNA lnASCs, control shRNA obASCs, and leptin shRNA obASCs.

### Stable transfection of shRNA

shRNA constructs targeting leptin and an shRNA construct targeting a non-human gene serving as a negative control were purchased from SA Biosciences (Frederick, MD, USA). The GFP sequence in the shRNA construct was removed and replaced with dsRed and neomycin resistance, producing a dsRed, neomycin-resistant leptin shRNA construct and a dsRed, neomycin-resistant negative control shRNA construct. The lnASCs (*n* = 6 donors) and obASCs (*n* = 6 donors) were transfected with a dsRed, neomycin-resistant leptin shRNA construct or a dsRed, neomycin-resistant negative control shRNA construct using the Neon Transfection System (Invitrogen), using 1400 V for the pulse voltage, 10 ms for the pulse width, and three pulses. Cells were allowed to recover, expanded, underwent antibiotic selection for 2 weeks, and sorted by flow cytometry to verify dsRed expression. Four groups of cells (*n* = 6 donors/group) were produced: control shRNA lnASCs, leptin shRNA lnASCs, control shRNA obASCs, and leptin shRNA obASCs.

### Alamar blue cell proliferation assay

Alamar blue cell proliferation assay was conducted according to manufacturer’s instructions. Briefly, 100 cells from each donor (lnASCs, control shRNA lnASCs, leptin shRNA lnASCs, obASCs, control shRNA obASCs, or leptin shRNA obASCs) were plated in a 96-well plate in triplicates. After cells had adhered overnight, the medium was removed, the wells were washed three times in PBS, and the cells were incubated in 10 % Alamar Blue reagent (Invitrogen). After 18 hours, the fluorescence intensity was measured at an excitation wavelength of 540 nm and an emission wavelength of 580 nm using a fluorescence plate reader. Cells were assessed on days 1, 3, and 7.

### RNA isolation followed by reverse transcriptase polymerase chain reaction (qRT-PCR)

Subconfluent cultures of control shRNA lnASCs (*n* = 6 donors), leptin shRNA lnASCs (*n* = 6 donors), control shRNA obASCs (*n* = 6 donors), and leptin shRNA obASCs (*n* = 6 donors) were analyzed. RNA was extracted from ASCs using TRIzol reagent (Invitrogen), purified with RNeasy columns (Qiagen, Valencia, CA, USA), and digested with DNase I (Invitrogen). A total of 1 μg of cellular RNA was used for cDNA synthesis with SuperScript VILO cDNA synthesis kit (Invitrogen). Quantitative real-time PCR was performed using the EXPRESS SYBR GreenER qPCR SuperMix Kit (Invitrogen) according to the manufacturer’s instructions. The following primer set sequence for leptin (forward 5′-gaagaccacatccacacacg-3′, reverse 5′-agctcagccagacccatcta-3′) and β-actin (forward 5′-caccttctacaatgagctgc-3′ and reverse 5′-tcttctcgatgctcgacgga-3′) were used. At the completion of the reaction, ΔΔ cycle threshold (ΔΔ C_t_) was calculated to quantify mRNA expression.

### Characterization of ASCs

ASCs were characterized as previously described [17]. Briefly, ASCs were induced to undergo osteogenic or adipogenic differentiation. For osteogenic differentiation, ASCs were cultured in 6-well plates in CCM until 70 % confluent and medium was replaced with fresh medium containing osteogenic supplements, consisting of 50 μM ascorbate 2-phosphate (Sigma), 10 mM β-glycerol phosphate (Sigma), and 10 nM dexamethasone. After 3 weeks, cells were fixed in 10 % formalin for 1 hour at 4 °C and stained for 10 minutes with 40 mM Alizarin Red (pH 4.1; Sigma) to visualize calcium deposition in the extracellular matrix. Images were acquired at × 4 magnification using the Nikon Eclipse TE200 (Melville, NY, USA) with the Nikon Digital Camera DXM1200F and the Nikon ACT-1 software version 2.7. For adipogenic differentiation, ASCs were cultured in 6-well plates in CCM until 70 % confluent, and medium was replaced with fresh medium containing adipogenic supplements consisting of 0.5 μM dexamethasone (Sigma), 0.5 mM isobutylmethylxanthine (Sigma), and 50 μM indomethacin (Sigma). After three weeks, cells were fixed in 10 % formalin for 1 hour at 4 °C, stained for 10–15 minutes at room temperature with Oil Red O (Sigma) to detect neutral lipid vacuoles, and images were acquired at × 10 magnification.

To determine the ability to form colony-forming units (CFU), ASCs were plated at a density of 100 cells on a 10-cm^2^ plate in CCM and incubated for 14 days. Plates were rinsed three times with PBS, and 10 ml of 3 % crystal violet (Sigma) was added for 30 minutes at room temperature. Plates were washed three times with PBS and once with tap water.

Analysis by flow cytometry of the cell surface marker profile was conducted by harvesting ASCs with 0.25 % trypsin/1 mM EDTA for 3–4 minutes at 37 °C. A total of 3 × 10^5^ cells were concentrated by centrifugation at 500 × g for 5 minutes, suspended in 50 μl PBS and labeled with the primary antibodies. The following primary antibodies were used: anti-CD45-PeCy7, anti-CD11b-PeCy5, anti-CD166-PE, anti-CD105-PE, anti-CD90-PeCy5, anti-CD34-PE, isotype-control fluorescein isothiocyanate (FITC) human IgG1 and isotype-control PE human IgG2a were purchased from Beckman Coulter (Indianapolis, IN, USA). Anti-CD44-APC was purchased from BD Biosciences (Franklin Lakes, NJ, USA). The samples were incubated for 30 minutes at room temperature, washed with PBS, and analyzed with the Galios Flow Cytometer (Beckman Coulter, Brea, CA, USA), running Kaluza software (Beckman Coulter). To assay cells by forward and side scatter of light, the FACScan was standardized with microbeads (Dynosphere uniform microspheres, Bangs Laboratories Inc., Thermo Scientific, Waltham, MA, USA). At least 10,000 events were analyzed and compared with isotype controls.

### BCC and ASC co-culture

BCCs were co-cultured with lnASCs (*n* = 6 donors) or obASCs (*n* = 6 donors) at 200 cells/cm^2^ in a 1:1 ratio in DMEM supplemented with 10 % FBS (Atlanta Biologicals) and P/S. After 7 days, cells were harvested, washed, and analyzed by flow cytometry. The percentage of GFP^+^ cells (BCCs) was determined using the Galios Flow Cytometer, running Kaluza software, and calculated based on the total number of cells. Where indicated, control shRNA lnASCs (*n* = 6 donors), leptin shRNA lnASCs (*n* = 6 donors), control shRNA obASCs (*n* = 6 donors), and leptin shRNA obASCs (*n* = 6 donors) were co-cultured with MCF7, ZR75, or T47D for 7 days. The percentage of GFP^+^ cells (BCCs) was determined with the Galios Flow Cytometer, running Kaluza software, and calculated based on the total number of cells. For RNA isolation, BCCs were sorted after co-culture with the Becton-Dickinson FACSVantage SE Cell Sorter with the DiVa option (BD, Franklin Lakes, NJ, USA) and analyzed with the DiVa software v5.02 (BD).

### Transwell migration and invasion assays

Migration assays were performed in transwell inserts with 8-μm-pore membrane filters, while invasion assays were performed with 8-μm transwell inserts pre-coated with a growth factor-reduced Matrigel layer to mimic basement membranes (BD Biosciences). BCC cells were cultured alone or co-cultured with control lnASCs, leptin shRNA lnASCs, control obASCs, or leptin shRNA obASCs for 7 days in a 1:1 ratio. BCCs were purified with FACS, and 1.25 × 10^4^ BCCs suspended in 50 μl were added to the apical chamber. A total of 200 μl of chemoattractant (10 % FBS; Atlanta Biologicals) was added to the basal chamber and incubated for 4 hours or 24 hours for migration or invasion, respectively. After the allotted time, the lower side of the transwell insert was carefully washed with cold PBS and non-migrating or non-invading cells remaining on the top chamber were removed with a cotton tip applicator. Migrating and invading cells were stained with Calcein-AM (2 μg/ml; Invitrogen) and measured on a fluorescent plate reader (FLUOstar optima, BMG Labtech Inc., Durham, NC, USA). Data were normalized to the respective BCCs without previous exposure to ASCs.

### RNA isolation followed by custom RT^2^ Profiler™ PCR arrays

To assess cells with the PCR array, total cellular RNA was extracted using RNeasy Mini Kit from FACS-purified BCCs cultured alone or after co-culture with a pool of control shRNA lnASCs, leptin shRNA lnASCs, control shRNA obASCs, or leptin shRNA obASCs, with six donors per group (Qiagen). RNA was treated with DNase I (Qiagen) according to manufacturer’s instructions: 1 μg of RNA was converted to cDNA with the RT^2^ First Strand Kit (SA Biosciences) according to the manufacturer’s protocol. Where indicated, total cellular RNA was extracted from tumors using RNeasy Mini Kit, treated with DNase I, and converted to cDNA with the RT^2^ First Strand Kit according to the manufacturer’s instructions. Gene expression profiling was performed using a Custom Breast Cancer RT^2^ Profiler PCR Array (SA Biosciences) and RT^2^ qPCR Master Mix (SA Biosciences). The Custom Breast Cancer RT^2^ Profiler PCR Array was manufactured to detect the expression of the following genes: *SERPINE1, IGFBP3, GSTP1, MMP-2, SNAI2, IL-6, PGR, TWIST1, PTGS2, SFRP1, THBS1, CDKN2A, PLAU, CSF1*, and *ACTB*. PCR amplification was performed in a Bio-Rad CFX96 Real-Time System (Hercules, CA, USA). The reaction conditions were as follows: 95 °C for 10 minutes, 40 cycles of 95 °C for 15 sec and 60 °C for 1 minute, followed by a dissociation curve. At the completion of the reaction, C_t_ values were determined, and ΔΔ C_t_ and fold change were determined using the RT^2^ Profiler PCR Array Data Analysis web portal (SA Biosciences). Genes with mRNA levels increased or decreased more than two-fold were considered significantly differentially expressed (*P* <0.05).

### In vivo tumorigenicity assay

SCID/beige (CB17.Cg-Prkdc^scid^Lyst^bg-J^/Crl) immunocompromised female ovariectomized mice (5 weeks old) were obtained from Charles River Laboratories (Wilmington, MA, USA). To assess whether leptin impacts tumorigenicity, mice were divided into three groups (*n* = 5 mice/group): MCF7 cells only, MCF7 cells plus control shRNA obASCs (*n* = 6 donors), and MCF7 plus leptin shRNA obASCs (*n* = 6 donors). Estradiol pellets (0.72 mg, 60-day release, Innovative Research of America, Sarasota, FL, USA) were implanted subcutaneously in the lateral area of the neck. Cell implants were prepared with MCF7 cells (10^6^) alone or MCF7 cells (10^6^) in combination with ASCs (10^6^) suspended in a total volume of 150 μl (one part sterile PBS and two parts reduced growth factor Matrigel (BD Biosciences). Cells were injected subcutaneously into the fifth mammary fat pad on both sides. All procedures in animals were carried out under anesthesia using a mixture of isoflurane and oxygen delivered continuously by mask. After 36 days, animals were euthanized by cervical dislocation after exposure to CO_2_. Organs were removed, weighed, digitally imaged, and fixed in 10 % neutral buffered formalin. Where indicated, additional mice were divided into three groups (*n* = 5 mice/group): MCF7 only, MCF7 plus lnASCs, and MCF7 plus obASCs to assess for potential metastasis by lnASCs or obASCs. Lungs and livers were also harvested and fixed in 10 % neutral buffered formalin for histological analyses.

All procedures involving animals were conducted in compliance with State and Federal law, standards of the US Department of Health and Human Services, and guidelines established by Tulane University Institutional Animal Care and Use Committee (IACUC). All animal protocols were approved by the Tulane University IACUC.

### Flow cytometry

Flow cytometry was conducted on the tumors to assess for GFP expressing MCF7 cells and dsRed expressing ASCs. Tumors were dissociated with collagenase/hyaluronidase (Stem Cell Technologies, Vancouver, BC, Canada) for 16 hours at 37 °C. After enzymatic dissociation, the reaction was neutralized with pre-warmed medium consisting of 10 % FBS (Atlanta Biologicals). Cells were centrifuged at 350 × g for 10 minutes, counted, and resuspended in PBS. The samples were then analyzed with the Galios Flow Cytometer running Kaluza software.

### Immunohistochemistry

Formalin-fixed, paraffin-embedded (FFPE) tumor, lung, and liver sections were de-paraffinized, rehydrated in Sub-X (Leica, Buffalo Grove, IL, USA) and graded solutions of ethanol, and stained with hematoxylin and eosin. FFPE tumor sections were de-paraffinized, rehydrated in Sub-X and graded solutions of ethanol, quenched with 0.3 % H_2_O_2_ (Sigma), rinsed with Tris-NaCl-Tween buffer (TNT), which consisted of 0.1 M Tris-HCl (pH 7.5; Sigma), 0.15M NaCl (Sigma), and 0.05 % Tween-20 (Invitrogen). Tumor sections were then blocked with 1 % BSA, and stained with primary antibodies obtained from Abcam against GFP, dsRed, SERPINE1, or matrix metalloproteinase-2 (MMP-2) overnight at 4 °C. Each tumor section was subsequently washed in TNT buffer. Tissue sections were incubated with appropriate horseradish peroxidase (HRP)-conjugated secondary antibody (Abcam) for 1 hour at room temperature, and washed with TNT buffer. For colorimetric staining, slides were then incubated in 3,3′-Diaminobenzidine (DAB; Vector Laboratories), washed with TNT, counterstained with hematoxylin (Thermo Scientific), and rinsed with deionized water. Slides were dehydrated in graded solutions of ethanol, followed by SubX in the final step, and sealed with Permount Mounting Medium (Sigma). After staining, images were acquired at × 10 and × 40 magnification with the ScanScope CS2 (Aperio, Vista, CA, USA).

### Protein isolation and western blot

Protein lysates were isolated with radioimmunoprecipitation assay (RIPA) buffer (Pierce; Thermo Scientific) from primary tumors formed with MCF7 cells, MCF7 cells mixed with control shRNA obASCs, or MCF7 cells mixed with leptin shRNA obASCs. Tumors were homogenized in RIPA buffer for 5 minutes, and the cell lysate was clarified by centrifugation at 15,000 × g for 15 minutes. Protein concentration was determined by the BCA Protein Assay (Pierce). Lysate (20 μg) was resolved on 4−12 % SDS-polyacrylamide gels and transferred to nitrocellulose membranes (Invitrogen). Blots were blocked with blØk Noise Canceling Reagents (Millipore Billerica, MA, USA). Blots were then incubated with anti-leptin antibody (R&D Systems; Minneapolis, MN, USA) overnight at 4 °C and washed with PBS with Tween 20 three times before being incubated with species-specific IgG conjugated to HRP for 1 hour at room temperature. Antigen-antibody complexes were visualized after incubation in chemiluminescence reagent (Invitrogen). Blots were imaged on an ImageQuant LAS 4000 (GE Healthcare Life Sciences; Piscataway, NJ, USA) and quantitative analysis of western blots was performed with densitometry.

### Metastasis assessment

Metastatic lesions were quantified by determining the area occupied by the lesion divided by the total area of the tissue section. The percentage the tissue occupied by metastatic cells in the liver and lung were averaged together for each mouse (*n* = 5 mice/group) and represented as the metastatic index.

### Statistical analysis

All values are presented as means ± standard error of the mean (SEM). The statistical differences among three or more groups were determined by analysis of variance (ANOVA), followed by post-hoc Tukey multiple comparison tests versus the respective control group. Statistical significance was set at *P* <0.05. Analysis was performed using Prism (Graphpad Software, San Diego, CA, USA).

## Results

### obASCs enhance ER^+^ BCC proliferation

**Fig. 1:**
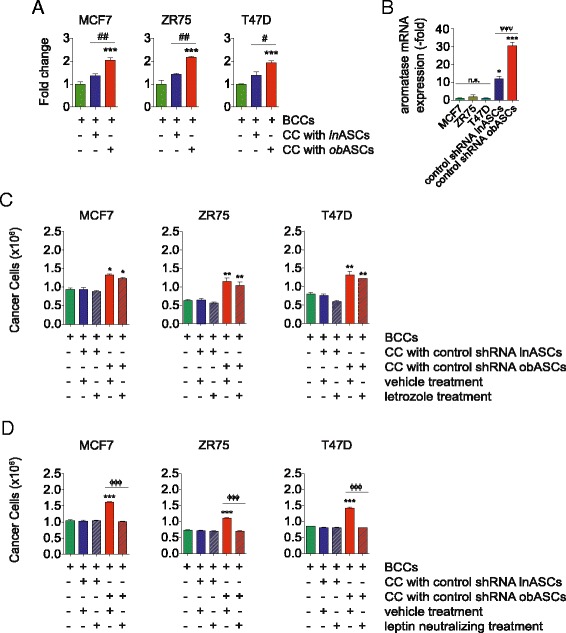
Adipose stromal/stem cells (ASCs) isolated from obese women (*obASCs*) enhance breast cancer cell (*BCC*) proliferation. **a** Estrogen receptor positive BCCs were co-cultured (CC) with ASCs isolated from lean women (*lnASCs*) (*n* = 6 donors) or obASCs (*n* = 6 donors) for 7 days and FACS performed based on green fluorescent protein (GFP) expression. BCCs sorted by FACS were counted and data are shown relative to the number of cells cultured alone. **b** RNA was collected from MCF7 cells, T47D cells, ZR75 cells, control short hairpin RNA (*control shRNA*) lnASCs, and control shRNA obASCs and analyzed for aromatase expression by quantitative RT-PCR. Data were normalized to the β-actin expression. **c** Conditioned media (CM) were collected from control shRNA lnASCs or control shRNA obASCs treated with vehicle or letrozole. BCCs were cultured in the CM for 7 days and the number of GFP^+^ BCCs was counted. **d** CM were collected from control shRNA lnASCs and control shRNA obASCs treated with vehicle or leptin neutralizing antibody. BCCs were cultured in the CM for 7 days and the number of GFP^+^ BCCs was counted. *Bar* represents ± standard error of the mean. *P <0.05, **P <0.01, ***P <0.001, relative to no co-culture BCCs; ^#^ *P* <0.05, ^##^ *P* <0.01 between BCCs co-cultured with lnASCs and obASCs; ^**ψψψ**^ *P* <0.001 relative to lnASCs and obASCs; ^ΦΦΦ^ *P* <0.001 between control shRNA obASCs treated with vehicle and leptin neutralizing antibody

The impact of ASCs on BCC growth was investigated using a co-culture assay. BCC lines MCF7, ZR75, and T47D were cultured alone or at a 1:1 ratio with either lnASCs or obASCs for 7 days. Prior to co-culture, BCCs were transduced with lentivirus expressing GFP in order to isolate BCCs expressing the fluorochrome following the co-culture period. obASCs increased the proliferation of ER^+^ BCCs to 2.0 ± 0.2 fold in MCF7 cells, 2.2 ± 0.1 fold in ZR75, and 1.9 ± 0.1 fold in T47D (*P* <0.05; Fig. 1a). While lnASCs increased the proliferation of ER^+^ BCCs, the effect was not as robust and was not statistically significant (Fig. 1a). These results suggest that ASCs increase the proliferation of all BCCs; however, obASCs markedly increased the proliferation of ER^+^ BCCs over lnASCs.

Previously, our group demonstrated that obASCs produce high levels of the adipokine leptin. As aromatase is a downstream target of leptin, to investigate whether the effects of leptin were through increased aromatase activity, we determined the level of aromatase expression in lnASCs and obASCs and the effects of aromatase inhibitor on our system. The mRNA expression of aromatase was 12.1-fold and 30.4-fold higher in lnASCs and obASCs, respectively, compared to MCF7 cells (*P* <0.05; Fig. 1b). There was no significant difference in aromatase expression in cancer cells (Fig. 1b). To assess the functional activity of aromatase in our system, we collected CM from ASCs treated with vehicle or letrozole. CM collected from vehicle-treated obASCs induced proliferation of MCF7 cells (by 53.4 %), ZR75 cells (by 83.9 %) and T47D cells (by 65.2 %; *P* <0.05; Fig. 1c). Upon treatment with CM collected from lnASCs and obASCs treated with letrozole, a reduction in cancer cell number was observed; however, cancer cells exposed to CM from obASCs treated with letrozole were still able to induced BCC proliferation. These results suggest that while aromatase produced by obASCs may contribute to increased proliferation of cancer cells, alternative pathways activated by leptin may be driving the effects of obASCs on BCCs proliferation. Furthermore, to determine whether leptin secreted by obASCs alone enhances breast cancer cell proliferation, CM from lnASCs and obASCs treated with leptin neutralizing antibody were exposed to BCCs. CM from obASCs treated with leptin neutralizing antibody displayed reduced proliferative potential on BCCs (1.0 × 10^6^ in MCF7 cells, 0.7 × 10^6^ in ZR75 cells, and 0.8 × 10^6^ in T47D cells), compared to CM from obASCs treated with vehicle (1.6 × 10^6^ in MCF7 cells, 1.1 × 10^6^ in ZR75 cells, and 1.4 × 10^6^ in T47D cells; Fig. 1d). CM from lnASCs treated with vehicle or leptin neutralizing antibody demonstrated similar results (Fig. 1d). Collectively, these results indicate that leptin plays a significant role in increasing BCC proliferation and indicate that leptin alone in this system enhances the proliferation of BCCs.

### obASCs expressing leptin shRNA have diminished capacity to enhance BCC proliferation

The role that leptin plays in the biology of obASCs and their impact on BCCs was investigated through the stable transfection of shRNA constructs targeting leptin into lnASCs and obASCs. These cells were used to determine the impact of silencing leptin on BCC proliferation. Leptin knockdown lnASCs and obASCs were initially collected to demonstrate a reduction of leptin expression and functional assays were performed to determine changes in stem cell properties. Knockdown efficiency was assessed with real-time RT-PCR after purification of transfected cells with FACS. lnASCs showed a reduction in leptin expression by 63.8 %, from 1.04-fold in control shRNA lnASCs to 0.37-fold in leptin shRNA lnASCs, and obASCs demonstrated a reduction in leptin expression by 99.4 %, from 147.81-fold in control shRNA obASCs to 0.85-fold in leptin shRNA obASCs, respectively (*P* <0.05; Additional file 1). No statistically significant difference was observed in the transcript level of leptin between leptin shRNA lnASCs and leptin shRNA obASCs. To characterize the cells, lnASCs or obASCs transfected with the leptin shRNA or control shRNA were differentiated in osteogenic differentiation medium or adipogenic differentiation medium, plated to determine colony forming unit capacity, and analyzed for the expression of cell surface markers. Transfected ASCs did not differ from non-transfected ASCs and maintained the ability to generate colony forming units and differentiate into bone and fat (Additional file 1). The cells also retained expression of CD44, CD90, CD105, and CD166 (while negative for the lymphohematopoietic markers CD34 and CD45 and monocyte marker CD11b; Additional file 1) similar to untransfected ASCs. The proliferation rate of transfected ASCs did not differ from non-transfected ASCs (Additional file 1), and no significant difference was observed between the proliferation rate of lnASCs and obASCs transfected with control shRNAs or leptin shRNAs (Additional file 1). The proliferative effects of CM collected from control shRNA lnASCs, leptin shRNA lnASCs, control shRNA obASCs, or leptin shRNA obASCs was assessed in BCCs. CM collected from control shRNA obASCs enhanced the number of BCCs, whereas CM collected from leptin shRNA obASCs did not display similar induction (Additional file 2).

**Fig. 2:**
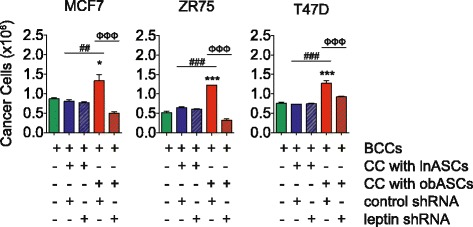
Leptin is essential for the adipose stromal/stem cells isolated from obese women (*obASC*)-driven breast cancer cell (*BCC*) proliferation. Adipose stromal/stem cells isolated from lean women (*lnASCs*) and obASCs transfected with either a control short hairpin RNA (*control-shRNA*) or leptin shRNA were co-cultured (CC) with BCCs. After 7 days, the green fluorescent protein-positive (GFP^+^) BCCs were sorted by FACS and counted. *Bar* represents ± standard error of the mean. *P <0.05, ***P <0.001, relative to no co-culture; ^##^P <0.01, ^###^ *P* <0.001 between BCCs co-cultured with control shRNA lnASCs and control shRNA obASCs; ^ΦΦΦ^ *P* <0.001 for comparison between BCCs co-cultured with control shRNA obASCs and leptin shRNA obASCs

The direct role of leptin secreted from obASCs on BCC proliferation was investigated by co-culturing control shRNA lnASCs, leptin shRNA lnASCs, control shRNA obASCs, or leptin shRNA obASCs with BCCs at a 1:1 ratio. The proliferation of BCCs was compared to BCCs cultured alone. MCF7, ZR75, and T47D cells demonstrated an increase in the total cell number following co-culture with control shRNA obASCs compared to BCCs cultured alone or co-cultured with control lnASCs (*P* <0.05; Fig. 2). The obASCs transfected with the leptin shRNA were unable to induce proliferation of BCCs in comparison to the obASC expressing the control shRNA. Leptin shRNA obASCs reduced the extent of proliferation in MCF7, ZR75, and T47D from 1.3E6 cells to 0.5E6 cells (2.2-fold decrease), 1.2E6 cells to 0.3E6 cells (4.0-fold decrease), and 1.3E6 cells to 0.9E6 cells (1.4-fold decrease), respectively, comparing BCCs exposed to control shRNA obASCs to leptin shRNA obASCs (*P* <0.05; Fig. 2). To assess whether CM collected from ASCs was able to induce BCC proliferation, CM was collected from control shRNA lnASCs, leptin shRNA lnASCs, control shRNA obASCs, and leptin shRNA obASCs. BCCs incubated in control shRNA obASCs induced BCC proliferation by 1.4-fold in MCF7 cells, 1.8-fold in ZR75 cells, and 1.6-fold in T47D cells; however BCCs incubated in control shRNA lnASCs, leptin shRNA lnASCs, and leptin shRNA obASCs were unable to induce BCC proliferation. These results implicate obASC-derived leptin as a key factor leading to enhanced proliferation of BCCs.

### obASCs enhance BCC migration and invasion

**Fig. 3:**
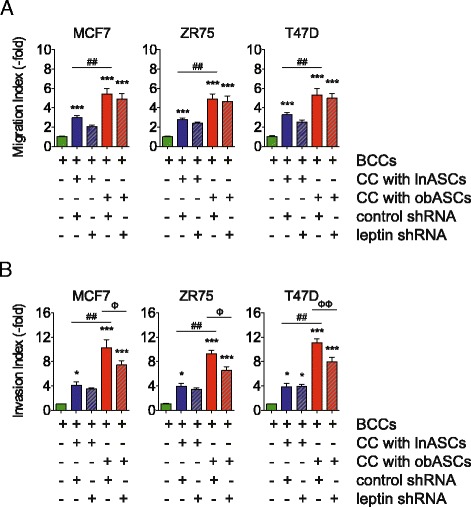
Adipose stromal/stem cells isolated from obese women (*obASC*)-derived leptin increases invasion of breast cancer cells (*BCCs*). BCC cells were co-cultured (CC) with adipose stromal/stem cells isolated from lean women (*lnASCs*) or obASCs stably transfected with a control short hairpin RNA (*control shRNA*) or leptin shRNA (leptin shRNA) for 7 days. Green fluorescent protein-positive BCC cells were isolated with FACS and plated in the top chamber of **a**, an uncoated 8-μm pore membrane, and assessed after 4 hours for migratory potential or **b**, a Matrigel-coated 8-μm pore membrane and assessed after 24 hours for invasive capacity. Medium containing FBS was plated in the lower chamber and served as a chemoattractant. Data are shown relative to cells without prior exposure to lnASCs or obASCs. *Bar* represents ± standard error of the mean: *P <0.05, ***P <0.001 relative to BCCs cultured alone; ^##^ *P* <0.01 for comparison between BCCs after co-culture with control shRNA lnASCs and control shRNA obASCs; ^Φ^ *P* <0.05, ^ΦΦ^ *P* <0.01 between BCCs after co-culture with control shRNA obASCs and letpin shRNA obASCs

BCCs were directly co-cultured for 7 days with lnASCs or obASCs transfected with control shRNA or leptin shRNA and collected by detection of GFP expression using FACS. Sorted cells were re-suspended in serum-free medium and plated on top of a transwell and assessed for migration after 4 hours or plated on top of a Matrigel-coated transwell and assessed for invasion after 24 hours. The BCCs collected after co-culture demonstrated enhanced migration towards chemoattractants by at least 2.7-fold relative to non-co-cultured BCCs, following direct co-culture of BCCs with lnASCs or obASCs (Fig. 3a). Silencing of leptin slightly diminished the capacity of both lnASCs and obASCs, to affect BCC migration; however, these results were not statistically significant (Fig. 3a). The invasive capacity of BCCs was enhanced after direct exposure to lnASCs and obASCs. The impact of obASCs on BCC invasion was more robust than the effect of lnASC on BCC invasion. lnASCs enhanced the invasion of MCF7, ZR75, and T47D cells by 4.1 ± 1.1, 3.9 ± 0.8, and 3.8 ± 1.0 times, respectively, while obASCs increased the invasive potential of MCF7, ZR75, and T47D cells by 10.2±2.3, 9.2±1.1, and 11.0±1.2 times, respectively (*P* <0.05; Fig. 3b). Inhibiting leptin expression in obASCs significantly decreased the levels of BCC invasion, while reducing leptin expression in lnASCs did not have the same impact. Leptin inhibition in obASCs reduced MCF7, ZR75, and T47D cell invasion by 27.5 %, 29.3 %, and 28.1 %, respectively (*P* <0.05; Fig. 3b). These results suggest that while obASCs have a greater capacity to enhance the migration and invasion of BCCs compared to lnASCs, obASC-derived leptin only plays an integral role in BCC invasion.

### Leptin shRNA obASCs reduce expression of proliferation and epithelial-to-mesenchymal transition (EMT) genes

The mechanism(s) by which ASC-derived leptin affects BCC signaling pathways was investigated using a custom breast cancer PCR array to determine changes in the expression of previously identified cell cycle, apoptotic, and angiogenic genes. Previously, our group showed alterations in these genes in the MCF7 cells after co-culture with obese ASCs [13]. Of the cell cycle, apoptotic, and angiogenic genes assessed (*CDKN2A, GSTP1, SFRP1, PLAU, THBS1, CSF*), none were significantly increased in the three BCC lines following direct co-culture with obASCs (Additional files 3, 4 and 5).

**Fig. 4:**
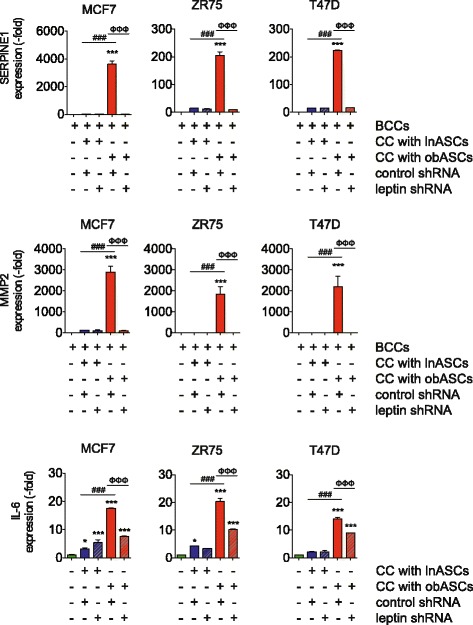
Leptin inhibition reduces the expression of key regulatory genes involved in invasion and metastasis. Breast cancer cells (*BCCs*) were co-cultured CC with adipose stromal/stem cells isolated from lean women (*lnASCs*) or adipose stromal/stem cells isolated from obese women (*obASCs*) stably transfected with a control short hairpin RNA (*control shRNA*) or leptin shRNA. After 7 days, co-cultured cells were sorted by FACS and BCCs were collected for total RNA extraction and cDNA synthesis. Genes involved in invasion and metastasis were assessed. *Bar* represents ± standard error of the mean. *P <0.05, ***P <0.001 relative to BCCs cultured alone; ^###^ *P* <0.01 for comparison between BCCs after co-culture with control shRNA lnASCs and control shRNA obASCs; ^ΦΦΦ^ *P* <0.001 for comparison between BCCs after co-culture with control shRNA obASCs and leptin shRNA obASCs. *MMP* matrix metalloproteinase

In contrast, investigation of EMT and metastatic genes previously identified by us to be altered in MCF7 cells after co-culturing with obASCs showed uniform changes in all three BCC lines. Of the genes assessed, BCCs overexpressed *SERPINE1, IL-6*, and *MMP-2* following direct co-culture with obASCs. Furthermore, inhibition of leptin in obASCs diminished the expression of these genes in BCCs (relative to obese ASCs; *P* <0.05; Fig. 4; Additional files 3–5). No statistically significant differences in gene expression were observed in BCCs after co-culture with control shRNA lnASCs or leptin shRNA lnASCs (Fig. 4; Additional files 3–5). *SERPINE1* expression was significantly decreased in MCF7, ZR75, and T47D after leptin knockdown 393.9-fold, 21.7-fold, and 12.6-fold, respectively (*P* <0.05; Fig. 4; Additional files 3−5). Additionally, leptin inhibition in obASCs limited the induction of *IL-6* expression in BCC by the obASCs, with a 2.3-fold decrease in MCF7 cells, 2.0-fold decrease in ZR75, and 1.5-fold decrease in T47D (*P* <0.05; Fig. 4; Additional files 3–5). Moreover, inhibition of leptin in obASCs resulted in a 27.4-fold, 179.2-fold, and 843.5-fold decrease in *MMP-2* expression in MCF7, ZR75, and T47D cells, respectively (*P* <0.05; Additional files 3–5). Together, these results indicate that obASCs increase the mRNA expression of key metastatic genes in ER^+^ BCC lines and suggest that obASCs may play a significant role in EMT and metastasis.

### Leptin shRNA obASCs result in diminished tumorigenicity and expression of EMT genes

**Fig. 5:**
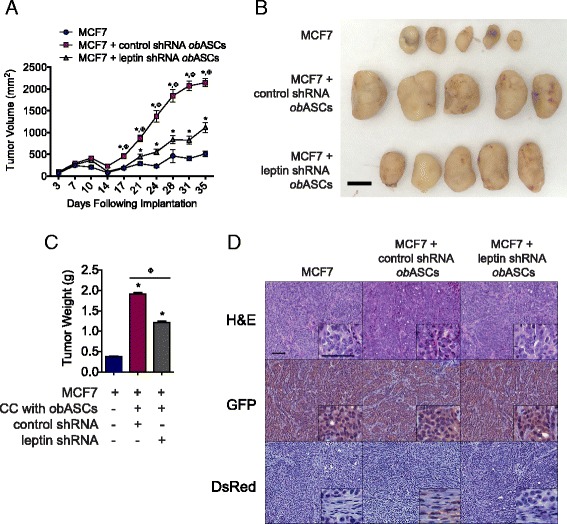
Leptin inhibition in adipose stromal/stem cells isolated from obese women (*obASCs*) reduces tumor volume. MCF7 cells were prepared alone or co-injected with control short hairpin shRNA (*control shRNA*) obASCs or leptin shRNA obASCs (1:1 ratio) into the mammary fat pad of SCID/beige mice (*n* = 5 mice/group). **a** Tumor volume was assessed every 3–4 days. **b** Representative tumors from each mouse (*n* = 5/group). *Scale bar* represents 1 cm. **c** Tumor weight on day 36. **d** Representative images of H&E, green fluorescent protein (*GFP*) staining, and dsRed staining of tumor sections visualized at × 10 and × 40 magnification (*inset*). *Scale bar* represents 50 μm. *Bar* represents ± standard error of the mean. *P <0.001 relative to MCF7 xenografts; ^Φ^ *P* <0.001 for comparison between MCF7 plus control shRNA obASC xenografts and MCF7 plus leptin shRNA obASC xenografts

In order to investigate the effects of leptin silencing on tumorigenesis, MCF7 cells were implanted alone or mixed with control shRNA obASCs or leptin shRNA obASCs into the mammary fat pad of SCID/beige mice. After 36 days, tumors formed with MCF7 cells mixed with leptin shRNA obASCs were significantly smaller (1115.1 ± 36.7 mm^3^ and 1.22 ± 0.03 g) compared to tumors formed with MCF7 cells mixed with control shRNA obASCs (2141.5 ± 29.5 mm^3^ and 1.92 ± 0.03 g) (*P* <0.001; Fig. 5a-c). Histological assessment of the tumors confirmed that the bulk of the tumor was composed of GFP expressing MCF7 cells (Fig. 5d). Furthermore, immunohistochemical analysis confirmed the presence of dsRed expressing ASCs in tumors formed with control shRNA obASCs mixed with MCF7 cells. In contrast, dsRed expression was not visible in tumors formed with MCF7 alone or mixed with leptin shRNA obASCs (Fig. 5d).

**Fig. 6:**
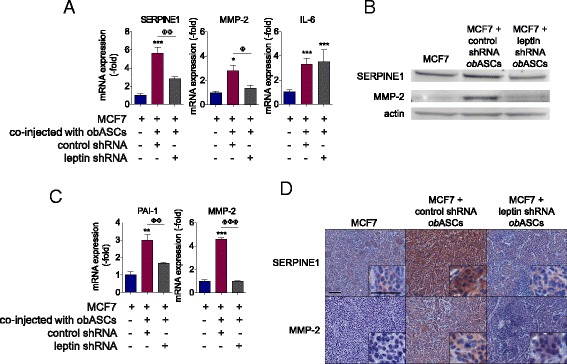
Adipose stromal/stem cells isolated from obese women (*obASC*)-derived leptin induces SERPINE1 and matrix metalloproteinase-2 (*MMP2*) expression in MCF7 xenografts. MCF7 cells were prepared alone or co-injected with control short hairpin RNA (*control shRNA*) obASCs or leptin shRNA obASCs (1:1 ratio) into the mammary fat pad of SCID/beige mice (n = 5 mice/group). After 36 days, tumors were harvested for analysis. **a** Total cellular RNA was isolated from tumors and analyzed for the expression of SERPINE1, MMP-2, and IL-6. **b** Western blot analysis of tumor lysate. A total of 20 μg of protein was separated by SDS-PAGE under reducing conditions, blotted, and probed with indicated antibodies. Blots were stripped and probed with actin antibody for normalization. **c** Densitometry analysis of SERPINE1 and MMP2 bands, normalized to actin. **d** Representative images of SERPINE1 staining and MMP-2 staining of tumor sections visualized at × 10 and × 40 magnification (*inset*). *Scale bar* represents 50 μm. *Bar* represents ± SD. *P <0.05, **P <0.01, ***P <0.0001 relative to MCF7 xenografts; ^Φ^ *P* <0.05, ^ΦΦ^ *P* <0.01, ^ΦΦΦ^ *P* <0.0001 between MCF7 plus control shRNA obASC xenografts and MCF7 plus leptin shRNA obASC xenografts

To assess the mechanism by which obASC-derived leptin affects BCC signaling in vivo, RNA was isolated from tumors and assessed with the custom breast cancer PCR array (Additional file 6). Assessment of the tumors showed increased expression of SERPINE1, MMP-2, and IL-6 in tumors formed with control shRNA obASCs mixed with leptin (*P* <0.05; Fig. 6a; Additional file 6). Inhibition of leptin in obASCs negated the gene induction of SERPINE1 and MMP-2 caused by obASCs in tumors. Western blot analysis was conducted on tumor samples to validate the expression of SERPINE1 and MMP-2 proteins. Tumors formed with MCF7 cells mixed with control shRNA obASCs demonstrated a 3.0-fold (±0.5) induction of SERPINE1 while tumors formed with MCF7 cells mixed with leptin shRNA obASCs demonstrated a 1.6-fold (±0.1) increase in SERPINE1, relative to MCF7 cells unexposed to ASCs (Fig. 6c). Likewise, tumors formed with MCF7 cells mixed with control shRNA obASCs demonstrated a 4.5-fold (±0.2) increase in MMP-2. Meanwhile, tumors formed with MCF7 cells mixed with leptin shRNA obASCs showed no difference from MCF7 cells without prior exposure to ASCs (Fig. 6c). Confirmation by immunohistochemical analysis demonstrated increased expression of SERPINE1 and MMP-2 in tumors formed with MCF7 cells and control shRNA ASCs compared to tumors formed with MCF7 cells and leptin shRNA ASCs (Fig. 6d).

### Leptin inhibition reduces obASC-induced metastasis of BCCs

**Fig. 7:**
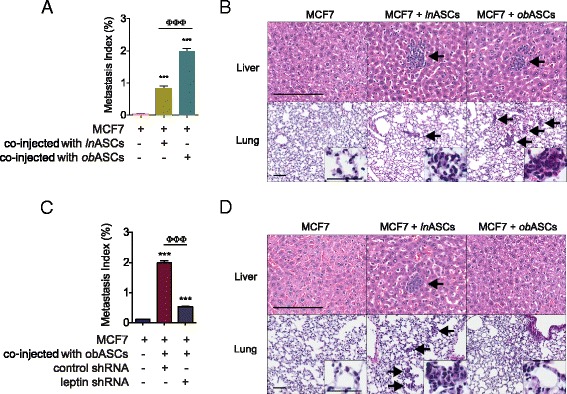
Leptin is essential for the adipose stromal/stem cells isolated from obese women (*obASC*)-driven metastasis MCF7 cells in vivo. **a** MCF7 cells were prepared alone or co-injected with adipose stromal/stem cells isolated from lean women (*lnASCs*) or obASCs (1:1 ratio) or **c**, co-injected with control short hairpin RNA (*control shRNA*) obASCs or leptin shRNA obASCs into the mammary fat pad of SCID/beige mice (n = 5 mice/group). Primary tumors were allowed to expand for 36 days, and the lung and liver were harvested for histological analysis of metastasis. Metastasis index was determined by the percentage of the liver and lung occupied by metastatic lesions. **b**, **d** Representative images of liver and lung sections. Liver sections were visualized at × 40 magnification and lung sections were visualized at × 10 and × 40 magnification (*inset*). *Scale bars* represent 50 μm. *Bar* represents ± standard error of the mean. ***P <0.0001 relative to MCF7 xenografts; ^###^ *P* <0.0001 for comparison between MCF7 plus lnASC xenografts and MCF7 plus obASC xenografts; ^ΦΦΦ^ *P* <0.0001 for comparison between MCF7 plus control shRNA obASC xenografts and MCF7 plus leptin shRNA obASC xenografts. *Arrows* highlight metastatic lesions within the liver or lung

As obASCs enhance the expression of EMT and metastatic genes in vitro, the role obASCs play in EMT and metastasis of BCCs was assessed in vivo. Immunocompromised mice were implanted with MCF7 cells alone, MCF7 cells mixed with lnASCs, or with obASCs for 36 days. Metastatic lesions in the lung and liver were visualized by H&E staining and quantified. Mice implanted with MCF7 cells alone had no metastatic lesions, whereas mice injected with MCF7 cells mixed with lnASCs (0.8 ± 0.07 %) and mice injected with MCF7 cells mixed with obASCs (2.0 ± 0.09 %) had significantly greater numbers of metastatic lesions (*P* <0.05; Fig. 7a-b; Additional file 7). These results indicate that while both lnASCs and obASCs enhanced metastasis of MCF7 cells, obASCs induced metastasis to the lung and liver more efficiently compared to lnASCs.

The effects of inhibiting obASC-derived leptin on EMT and metastasis were investigated in vivo. SCID/beige mice were implanted with MCF7 cells alone, MCF7 cells mixed with control shRNA obASCs, or MCF7 cells mixed with leptin shRNA obASCs for 36 days. Metastasis to the lung and liver was visualized by H&E staining and quantified. Mice injected with MCF7 cells mixed with control shRNA obASCs (2.0 ± 0.20 %) had significantly more metastasis compared to mice injected with MCF7 cells mixed with leptin shRNA obASCs (0.5 ± 0.04 %) (*P* <0.05; Fig. 7c-d; Additional file 7). These results suggest that obASCs-derived leptin drives metastasis of MCF7 cells in the lung and liver.

## Discussion

The incidence of obesity has been steadily increasing over the past few decades. Obese and overweight individuals now account for more than two thirds of the adult population in the USA [18]. Much co-morbidity is associated with obesity, and a clear epidemiological association between obesity and the prevalence of numerous cancers has been established; one being breast cancer [19]. While the relative risk amongst studies varies from 1.5 to 2.5, there is consensus that there is an increased relative risk of breast cancer development in women with BMI >30 [20–22].

Recent studies investigating the role of adipose tissue on breast cancer, in particular the excessive accumulation of adipose tissue in obesity, implicate ASCs as a significant factor contributing to disease development [23]. ASCs are stromal/stem progenitor cells of mesenchymal origin and have been shown to travel through the blood to distant tumor sites where they differentiate into vascular pericytes or secrete growth factors that support the tumor microenvironment [24, 25]. Additional studies suggest that ASCs originating from remote fat depots have the potential to traffic to the tumor and promote tumor progression through the secretion of proteases and pro-angiogenic factors [15, 26]. In the present study, the impact of obASCs on several BCC lines was investigated. The data presented here indicate that obASCs enhance the proliferation of ER^+^ BCCs and the preferential impact of obASC on ER^+^ BCC lines suggest that ER^+^ BCCs are able to respond to factors produced by the obASCs, which are not produced by lnASCs. Furthermore, the data suggests that TNBC may not express receptors or that their signaling pathways do not respond to the factors produced by obASCs.

Previously, ASCs have been shown to be a source of the leptin in the adipose tissue, and this leptin produced by the ASCs has been shown to stimulate BCC proliferation [13]. Leptin, a growth factor produced within adipose tissue, primarily functions to maintain energy balance. It also plays an important role in cell growth and differentiation under normal physiological conditions [27]. Previous studies have also indicated that a leptin-leptin receptor signaling axis may crosstalk with the ER and enhance tumorigenesis and metastasis [28–31]. These findings are consistent with our current report of obASCs and their preferential role in increasing ER^+^ BCCs but not ER^−^ BCCs.

Obesity has been shown to result in hyperleptinemia [32]. Hyperleptinemia leads to an increase in breast cancer cell proliferation, migration, and invasion, which gives rise to more aggressive and metastatic tumor cells [33–41]. Several laboratories have confirmed that exogenously delivered leptin increases BCC proliferation at different concentrations (100–1,600 ng/ml) [33–40]. Previously, studies have shown that the mechanism by which leptin promotes the survival of cancer cells is through the activation of multiple signaling pathways, such as those involving mitogen-activated protein kinase (MAPK), Janus kinase 2-signal transducer and activator of transcription 3 (JAK2-STAT3) and phosphatidylinositol 3-kinase-protein kinase B (PI3K-AKT) [42, 43]. However, additional studies are necessary to determine the sources of leptin in the adipose tissue and whether leptin secreted by the obASCs utilizes similar signal transduction pathways to promote the survival of cancer cells.

The mechanism by which obASC-derived leptin promotes alterations to the biology of BCCs was investigated by inhibiting leptin expression by stably transfecting lnASCs and obASCs with a leptin shRNA construct. The data suggest that obASC-derived leptin enhances several central processes such as proliferation and metastasis of cancer cells that ultimately enhance the aggressiveness of breast cancer cells. However, the precise mechanism by which obASC-derived leptin enhances proliferation remains to be determined. Our assessment of several key proliferative factors demonstrated increased expression of CDKN2A and SFRP1 in MCF7 and ZR75 cells following co-culture with obASCs. Inhibiting leptin expression negated the increased expression of CDKN2A and SFRP1 in MCF7 and ZR75 cells even after co-culture, suggesting that the expression of these two genes are leptin-mediated; however, these results did not translate into the T47D cells, which indicate obASC is not signaling through CDKN2A and SFRP1 to increase the proliferation of T47D cells. Therefore, additional analysis utilizing RNA-sequencing or other global approaches to assess gene expression may be warranted in order to assess the broader impact of obASCs and obASC-derived leptin on the proliferation of all ER^+^ BCCs.

With respect to migration and invasion, obASCs enhanced migration and invasion of BCCs. However, inhibition of leptin only reduced the invasive potential of BCCs. These results suggest that while other factors secreted by obASCs enhance BCC migration, leptin plays an important role in BCC invasion. Studies have shown that migration is largely dependent on integrins and adhesion molecules, while invasion is dependent on the expression of proteases [44, 45]. Therefore, obASC-secreted leptin likely regulates the expression of proteases.

With respect to metastasis, leptin appears to signal primarily through SERPINE1 and MMP-2 [46, 47]. SERPINE1, a serine protease inhibitor, limits the activity of matrix metalloproteases in the extracellular matrix microenvironment [48]. Paradoxically, SERPINE1 is also involved in other molecular interactions, including binding to the extracellular matrix protein vitronectin and endocytosis receptors of the low-density lipoprotein receptor (LRP) family. Binding of SERPINE1 to vitronectin results in detachment of the tumor cell from the extracellular matrix (ECM), leading to enhanced mobility of the cells [49]. Likewise, MMP-2 expression correlates with increased metastasis and poorer clinical prognosis [50, 51]. Furthermore, the cells expressing MMP-2 are also important and indicative of the aggressiveness of the breast cancer [52]. Elevated expression of MMP-2 in cancer cells has been found to be associated with smaller tumors, while expression of MMP-2 by stromal cells has been associated with increased aggressiveness [52]. Furthermore, expression of IL-6 was similarly upregulated in BCCs following co-culture with obASCs. However, tumors formed with a mixture of leptin shRNA obASCs and BCCs did not demonstrate similar reduction in IL-6, which suggests that other cells within the tumor microenvironment may be contributing to the increase in IL-6. In particular, studies have shown that immune cells express high levels of IL-6 during the cancer progression [53, 54]. Nevertheless, the elevated expression of SERPINE1 and MMP-2 correlated with the increased incidence of metastatic lesions in the lung and liver of mice injected with a mixture of obASCs and cancer cells, compared to mice that received cancer cells alone or cancer cells mixed with lnASCs. Furthermore, the expression of SERPINE1 and MMP-2 was reduced in tumors formed with cancer cells and leptin shRNA obASCs, relative to tumors formed with cancer cells and control shRNA obASCs. These results suggest that leptin affects the overexpression of these key metastatic factors within the tumor.

## Conclusion

obASCs not only contribute to the primary tumor growth, but these cells also enhance metastasis through a leptin-mediated pathway(s) involving SERPINE1 and MMP-2. The data suggest that the ASCs, which are in close proximity to the cancer cells within a tumor, are a novel avenue by which obesity influences the biology of cancer cells. Additional studies investigating the cells that contribute to the tumor microenvironment and their interaction with the cancer cells in obese patients are necessary to provide additional targets that will delineate the development and progress of breast cancer in these patients. With respect to leptin, the results of these studies would suggest that in obese individuals leptin is a primary molecule driving changes in the BCC that result in enhanced tumorigenicity and metastasis.

## Additional files

Additional file 1:
**Adipose stromal/stem cells (** ***ASCs*** **) isolated from lean women (** ***lnASCs*** **) and ASCs isolated from obese women (** ***obAS*** **C** ***s*** **) were stably** **transfected with control short hairpin RNA (** ***ctrl-shRNA*** **) and leptin shRNA (** ***lep-shRNA*** **).** lnASCs (*n* = 6 donors) and obASCs (*n* = 6 donors) were transfected with a ctrl-shRNA construct targeting a non-human gene or a lep-shRNA construct targeting leptin. Cells underwent antibiotic selection, followed by FACS. ASCs stably transfected with the shRNA vectors were assessed with real-time polymerase chain reaction to quantitative leptin expression at the RNA level (A). B Stably transfected ASCs were characterized by self-renewal capacity demonstrated by colony forming unit assay. Cells seeded at low density were assessed after 14 days by staining with crystal violet. C Stably transfected ASCs were plated in 6-well dishes and exposed to bone differentiation medium and fat differentiation medium to determine their ability to undergo adipogenic and osteogenic differentiation. After 21 days, cells were fixed and stained with Oil Red O or Alizarin Red for adipogenic differentiation and osteogenic differentiation, respectively. Representative images of ASCs acquired at × 10 (adipogenic differentiation) and × 4 (osteogenic differentiation) are shown. D Stably transfected cells were assessed by flow cytometry for the expression of indicated cell surface markers. E The proliferation rate of lnASCs and obASCs were compared with stably transfected control shRNA lnASCs and control shRNA obASCs. F The proliferative rate of stably transfected control shRNA and leptin shRNA lnASCs and obASCs were compared. Bar is ± SD. ****P* <0.001 for comparison of ctrl-shRNA lnASC and ctrl-shRNA obASCs, ^###^ *P* <0.001 for comparison of ctrl-shRNA obASCs and lep-shRNA obASCs (TIFF 6836 kb) Additional file 2:
**Leptin in conditioned media is essential for the adipose stromal/stem cells (** ***ASCs*** **) isolated from obese women (** ***obASC*** **)-driven breast cancer cell (** ***BCC*** **) proliferation.** Conditioned media (*CM*) were collected from control short hairpin RNA (*shRNA*) ASCs isolated from lean women (*lnASCs*), leptin shRNA lnASCs, control shRNA obASCs, and leptin shRNA obASCs. BCCs were cultured in the CM for 7 days and the number of GFP^+^ BCCs was counted. **P* <0.05; ***P* <0.01 relative to unconditioned cells; ^ΦΦΦ^ *P* <0.001 for comparison between control shRNA obASCs and leptin shRNA obASCs (TIFF 3589 kb) Additional file 3:
**mRNA expression of MCF7 cells after exposure to leptin (lep) knockdown adipose stromal/stem cells (ASCs).** Data are shown as fold change relative to the respective breast cancer cell line without previous co-culture with ASCs. **P* <0.05; ^#^ *P* <0.01; ^¥^ *P* <0.001. *EMT* epithelial-to-mesenchymal transition, *lnASCs,* adipose stromal/stem cells isolated from lean women, *obASCs* adipose stromal/stem cells isolated from obese women (PDF 50 kb) Additional file 4:
**mRNA expression of ZR75 cells after exposure to leptin knockdown adipose stromal/stem cells (ASCs).** Data are shown as fold change relative to respective breast cancer cell line without previous co-culture with ASCs. **P* <0.05; ^#^ *P* <0.01; ^¥^ *P* <0.001. *lep* leptin, *ctrl* control, *lnASCs,* adipose stromal/stem cells isolated from lean women, *obASCs* adipose stromal/stem cells isolated from obese women, *EMT* epithelial-to-mesenchymal transition (PDF 50 kb) Additional file 5:
**mRNA expression of T47D cells after exposure to leptin knockdown adipose stromal/stem cells (ASCs).** Data are shown as fold change relative to respective breast cancer cell line without previous co-culture with ASCs. **P* <0.05; ^#^ *P* <0.01; ^¥^ *P* <0.001. *lep* leptin, *ctrl* control, *lnASCs,* adipose stromal/stem cells isolated from lean women, *obASCs* adipose stromal/stem cells isolated from obese women, *EMT* epithelial-to-mesenchymal transition (PDF 50 kb) Additional file 6:
**mRNA expression of xenografts formed with MCF7 cells and leptin knockdown adipose stromal/stem cells (ASCs).** Data are shown as fold change relative to respective breast cancer cell line without previous co-culture with ASCs. **P* <0.05; ^#^ *P* <0.05; ^¥^ *P* <0.05. *lep* leptin, *ctrl* control, *lnASCs,* adipose stromal/stem cells isolated from lean women, *obASCs* adipose stromal/stem cells isolated from obese women, *EMT* epithelial-to-mesenchymal transition (PDF 49 kb) Additional file 7:
**Adipose stromal/stem (ASCs) cells isolated from obese women (** ***obASCs*** **) enhance metastasis of breast cancer cells.** A MCF7 cells were prepared alone or co-injected with adipose stromal/stem cells isolated from lean women (*lnASCs*) or *ob*ASCs (1:1 ratio), or B co-injected with control short hairpin RNA (*ctrl-shRNA*) obASCs or leptin short hairpin RNA (*lep-shRNA*) obASCs into the mammary fat pad of SCID/beige mice (*n* = 5 mice/group). After 36 days, tissues were harvested for histological analysis of metastasis. Representative histological images of liver sections with and without highlighted metastatic cells are shown. Images were acquired at × 2 magnification. (TIFF 6938 kb)

## Abbreviations

AKT: kinase-protein kinase B
ASC: adipose stromal/stem cell
BCC: breast cancer cell
BMI: body mass index
BSA: bovine serum albumin
CCM: complete culture medium
CDKN2A: cyclin-dependent kinase inhibitor 2A
CFU: colony-forming unit
CM: conditioned media
CSF1: colony stimulating factor 1
DAB: 3,3′-Diaminobenzidine
DMEM: Dulbecco’s modified Eagle’s medium
EMT: epithelial-to-mesenchymal transition
ER+: estrogen receptor positive
FACS: fluorescence-activated cell sorting
FFPE: formalin-fixed, paraffin-embedded
GFP: green fluorescent protein
GSTP1: glutathione S-transferase pi 1
H&E: hematoxylin and eosin
HRP: horseradish peroxidase
IL-6: interleukin 6
JAK2: Janus kinase 2
leptin shRNA lnASCs: adipose stromal/stem cells isolated from lean women transfected with leptin short hairpin RNA
leptin shRNA obASCs: adipose stromal/stem cells isolated from obese women transfected with leptin short hairpin RNA
lnASCs: adipose stromal/stem cells isolated from lean women
LRP: low-density lipoprotein receptor
MAPK: mitogen-activated protein kinase
MMP-2: matrix metalloproteinase-2
obASCs: adipose stromal/stem cells isolated from obese women
PBS: phosphate-buffered saline
PI3K: phosphatidylinositol 3
PLAU: plasminogen activator, urokinase
qRT-PCR: quantitative reverse transcriptase polymerase chain reaction
SEM: standard error of the mean
SERPINE1: plasminogen activator inhibitor-1
SFRP1: secreted frizzled-related protein 1
shRNA: short hairpin RNA
SM: stromal medium
STAT3: signal transducer and activator of transcription 3
THBS1: thrombospondin 1
TNBC: triple-negative breast cancer
TNT: Tris-NaCl-Tween buffer
