# Supplementary figures and images for: Leptin produced by obese adipose stromal/stem cells enhances proliferation and metastasis of estrogen receptor positive breast cancers
Source: Breast Cancer Res. 2015 Aug 19;17(1):112. doi: 10.1186/s13058-015-0622-z (PMC4541745; doi:10.1186/s13058-015-0622-z)

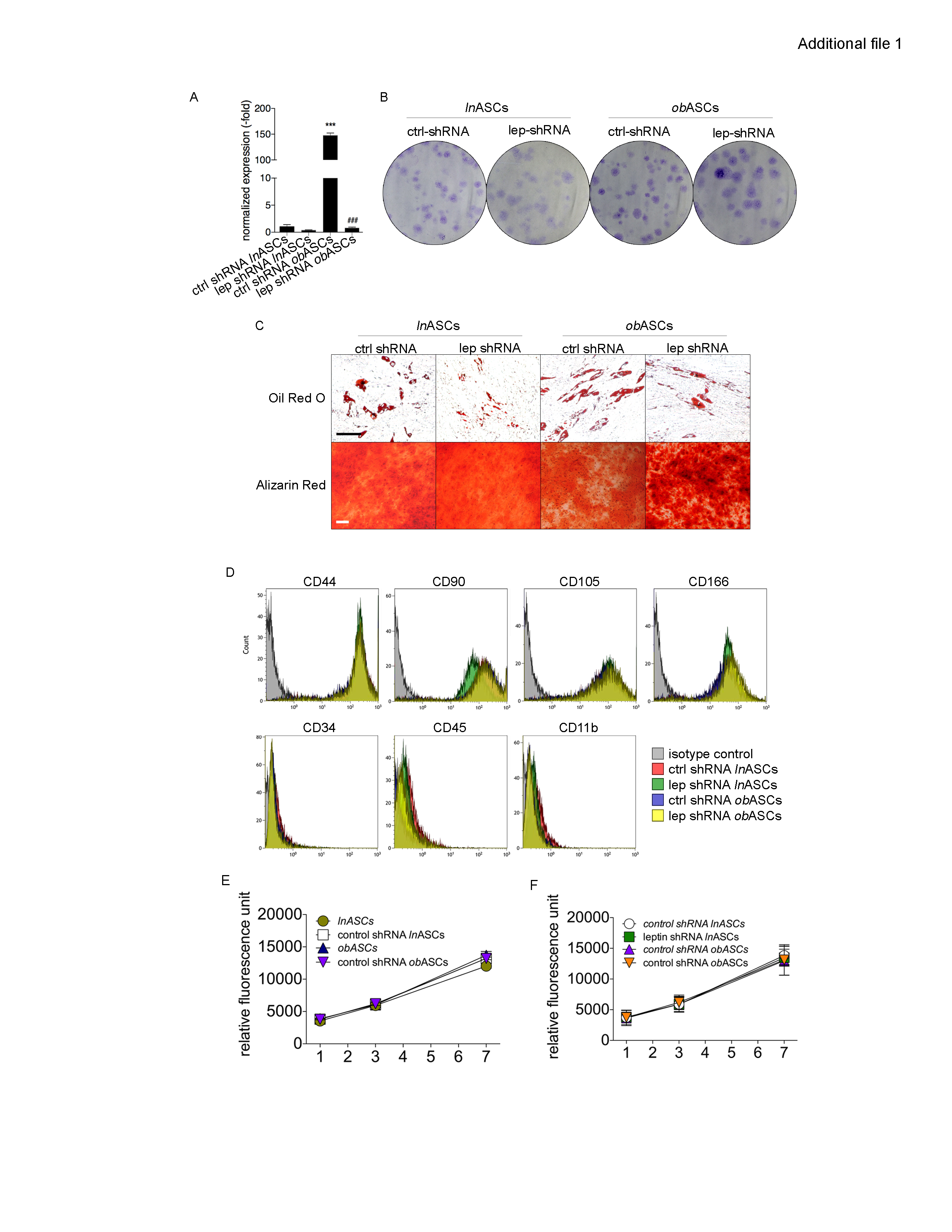

Supplement: Additional file 1: — Adipose stromal/stem cells ( ASCs ) isolated from lean women ( lnASCs ) and ASCs isolated from obese women ( obAS C s ) were stably transfected with control short hairpin RNA ( ctrl-shRNA ) and leptin shRNA ( lep-shRNA ). lnASCs (n = 6 donors) and obASCs (n = 6 donors) were transfected with a ctrl-shRNA construct targeting a non-human gene or a lep-shRNA construct targeting leptin. Cells underwent antibiotic selection, followed by FACS. ASCs stably transfected with the shRNA vectors were assessed with real-time polymerase chain reaction to quantitative leptin expression at the RNA level (A). B Stably transfected ASCs were characterized by self-renewal capacity demonstrated by colony forming unit assay. Cells seeded at low density were assessed after 14 days by staining with crystal violet. C Stably transfected ASCs were plated in 6-well dishes and exposed to bone differentiation medium and fat differentiation medium to determine their ability to undergo adipogenic and osteogenic differentiation. After 21 days, cells were fixed and stained with Oil Red O or Alizarin Red for adipogenic differentiation and osteogenic differentiation, respectively. Representative images of ASCs acquired at × 10 (adipogenic differentiation) and × 4 (osteogenic differentiation) are shown. D Stably transfected cells were assessed by flow cytometry for the expression of indicated cell surface markers. E The proliferation rate of lnASCs and obASCs were compared with stably transfected control shRNA lnASCs and control shRNA obASCs. F The proliferative rate of stably transfected control shRNA and leptin shRNA lnASCs and obASCs were compared. Bar is ± SD. ***P <0.001 for comparison of ctrl-shRNA lnASC and ctrl-shRNA obASCs, ### P <0.001 for comparison of ctrl-shRNA obASCs and lep-shRNA obASCs (TIFF 6836 kb) [file 13058_2015_622_MOESM1_ESM.tif]

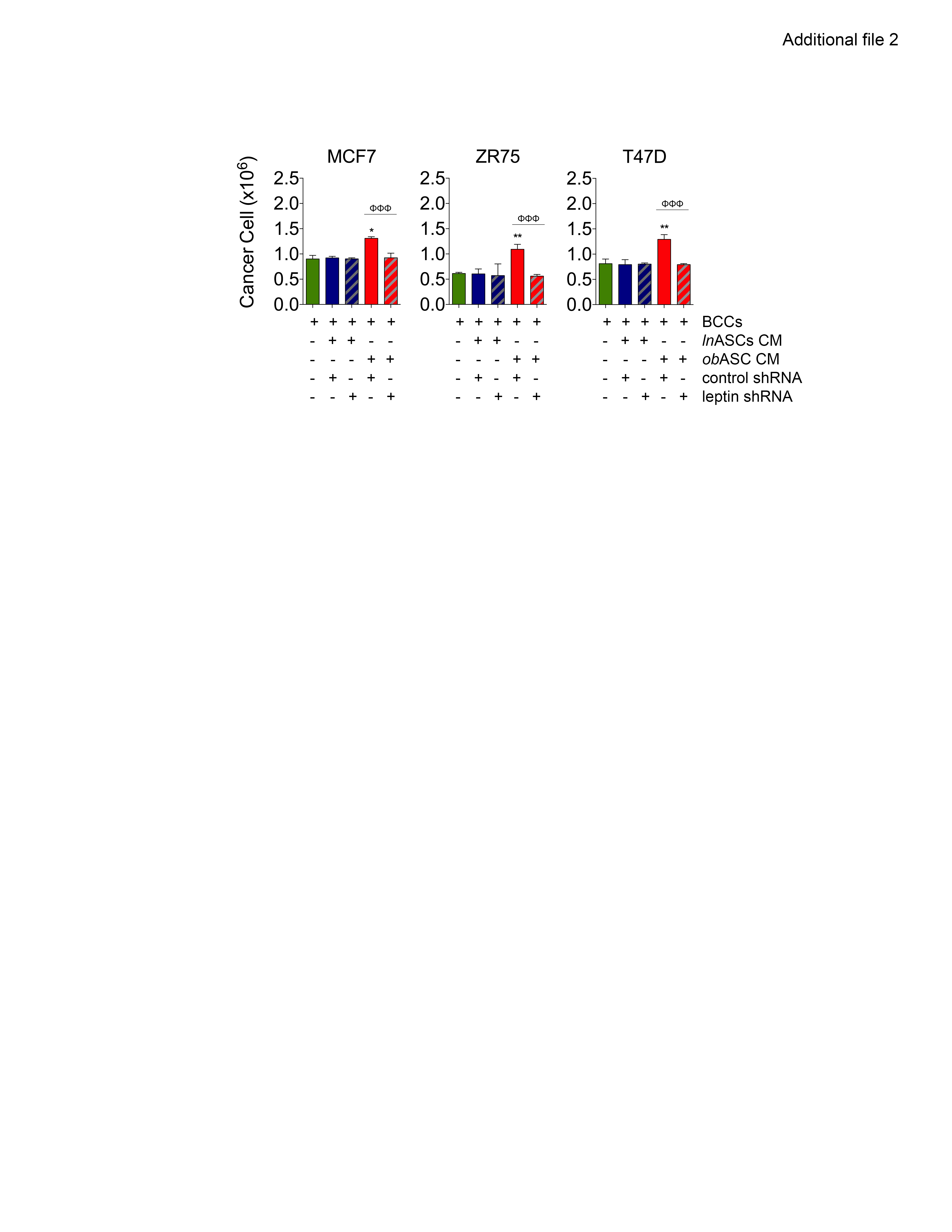

Supplement: Additional file 2: — Leptin in conditioned media is essential for the adipose stromal/stem cells ( ASCs ) isolated from obese women ( obASC )-driven breast cancer cell ( BCC ) proliferation. Conditioned media (CM) were collected from control short hairpin RNA (shRNA) ASCs isolated from lean women (lnASCs), leptin shRNA lnASCs, control shRNA obASCs, and leptin shRNA obASCs. BCCs were cultured in the CM for 7 days and the number of GFP+ BCCs was counted. *P <0.05; **P <0.01 relative to unconditioned cells; ΦΦΦ P <0.001 for comparison between control shRNA obASCs and leptin shRNA obASCs (TIFF 3589 kb) [file 13058_2015_622_MOESM2_ESM.tif]

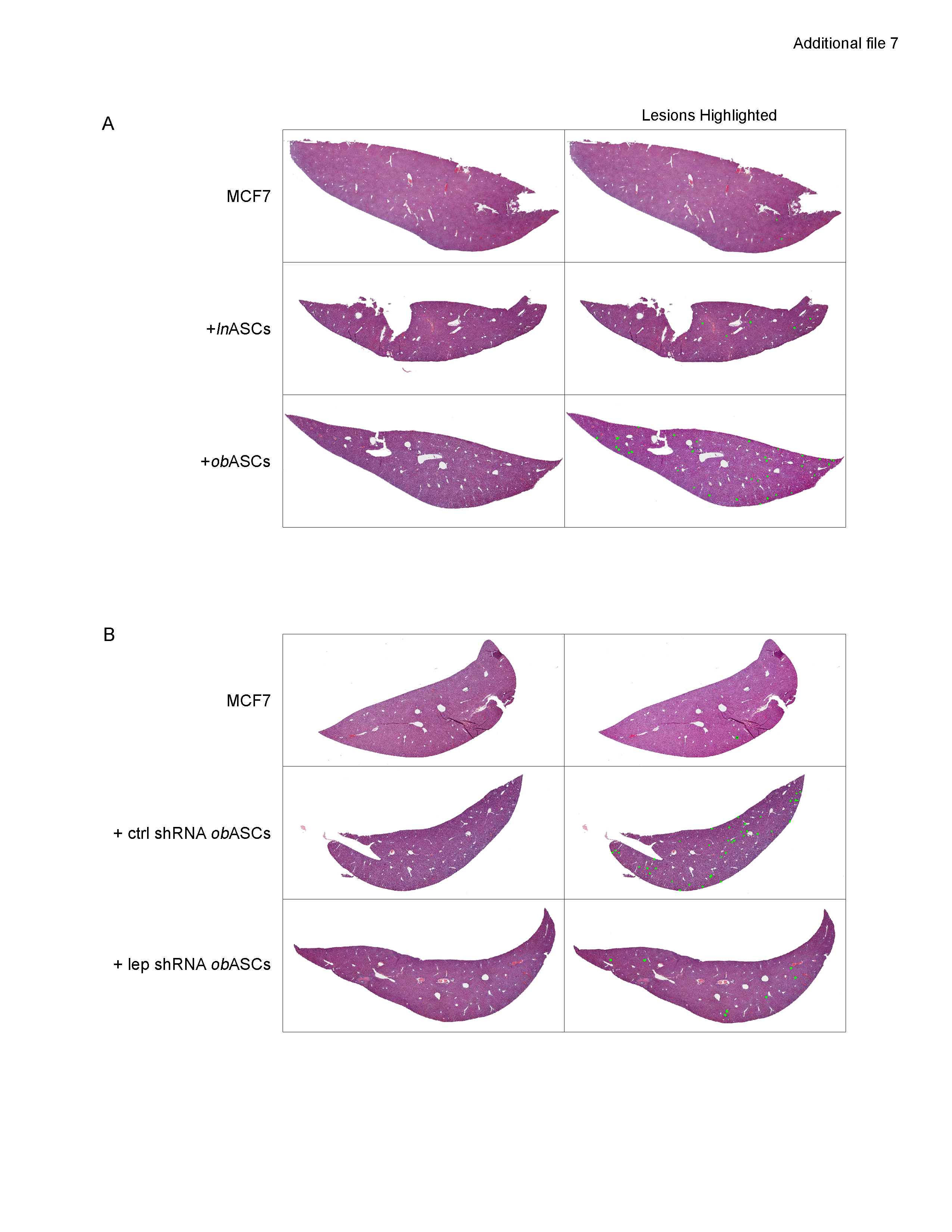

Supplement: Additional file 7: — Adipose stromal/stem (ASCs) cells isolated from obese women ( obASCs ) enhance metastasis of breast cancer cells. A MCF7 cells were prepared alone or co-injected with adipose stromal/stem cells isolated from lean women (lnASCs) or obASCs (1:1 ratio), or B co-injected with control short hairpin RNA (ctrl-shRNA) obASCs or leptin short hairpin RNA (lep-shRNA) obASCs into the mammary fat pad of SCID/beige mice (n = 5 mice/group). After 36 days, tissues were harvested for histological analysis of metastasis. Representative histological images of liver sections with and without highlighted metastatic cells are shown. Images were acquired at × 2 magnification. (TIFF 6938 kb) [file 13058_2015_622_MOESM7_ESM.tif]
