# Supplementary material for: Leptin produced by obese adipose stromal/stem cells enhances proliferation and metastasis of estrogen receptor positive breast cancers
Source: Breast Cancer Res. 2015 Aug 19;17(1):112. doi: 10.1186/s13058-015-0622-z (PMC4541745; doi:10.1186/s13058-015-0622-z)
Supplement: Additional file 3: — mRNA expression of MCF7 cells after exposure to leptin (lep) knockdown adipose stromal/stem cells (ASCs). Data are shown as fold change relative to the respective breast cancer cell line without previous co-culture with ASCs. *P <0.05; # P <0.01; ¥ P <0.001. EMT epithelial-to-mesenchymal transition, lnASCs, adipose stromal/stem cells isolated from lean women, obASCs adipose stromal/stem cells isolated from obese women (PDF 50 kb) [file 13058_2015_622_MOESM3_ESM.pdf]

		MCF7 cells after co-culture with ASCs			
Function	*Gene Name*	ctrl shRNA *ln*ASCs	lep shRNA *ln*ASCs	ctrl shRNA *ob*ASCs	lep shRNA *ob*ASCs
Cell Cycle and Apoptosis	*CDKN2A*	3.6	4.8	22.2[¥]	11.6*
	GSTP1	19.2*	20.7*	284.5[¥]	15.4*
	SFRP1	10.2*	1.0	74.4[¥]	19.9*
Angiogenesis	*PLAU*	1.4	1.3	5.9[¥]	2.1
	THBS1	16.4*	13.8*	20.9*	15.5*
	CSF	16.4[#]	0.3	20.9[¥]	15.5
EMT and Metastasis	*SERPINE1*	26.2*	29.9*	3624.0[¥]	9.2*
	MMP2	124.9	109.3	2895.0[¥]	105.7
	IL-6	3.2	5.5	17.6[¥]	7.6
	TWIST1	2.1	0.8	91.2[¥]	2.0
	PTGS2	3.2	9.5	81.0[¥]	54.5[#]
	SNAI2	2.2	4.2	517.0[¥]	4.2
