# Supplementary material for: Leptin produced by obese adipose stromal/stem cells enhances proliferation and metastasis of estrogen receptor positive breast cancers
Source: Breast Cancer Res. 2015 Aug 19;17(1):112. doi: 10.1186/s13058-015-0622-z (PMC4541745; doi:10.1186/s13058-015-0622-z)
Supplement: Additional file 4: — mRNA expression of ZR75 cells after exposure to leptin knockdown adipose stromal/stem cells (ASCs). Data are shown as fold change relative to respective breast cancer cell line without previous co-culture with ASCs. *P <0.05; # P <0.01; ¥ P <0.001. lep leptin, ctrl control, lnASCs, adipose stromal/stem cells isolated from lean women, obASCs adipose stromal/stem cells isolated from obese women, EMT epithelial-to-mesenchymal transition (PDF 50 kb) [file 13058_2015_622_MOESM4_ESM.pdf]

		ZR75 cells after co-culture with ASCs			
Function	*Gene Name*	ctrl shRNA *In*ASCs	lep shRNA *In*ASCs	ctrl shRNA *ob*ASCs	lep shRNA *ob*ASCs
Cell Cycle and Apoptosis	*CDKN2A*	2.4	1.9	10.2[#]	2.3
	GSTP1	3.3	3.1	0.8	4.0
	SFRP1	8.3*	3.1	50.3[#]	14.2*
Angiogenesis	*PLAU*	1.4	1.9	2.3*	1.2
	THBS1	1.2	1.6	2.3*	0.9
	CSF	1.4	1.4	2.3	1.9
EMT and Metastasis	*SERPINE1*	14.9*	12.0*	204.0[#]	9.4*
	MMP2	2.1	3.0	1845.9[¥]	10.3
	IL-6	4.2	3.3	20.3[¥]	10.2*
	TWIST1	3.5	2.6	15.0*	2.5
	PTGS2	1.3	1.1	2.3	2.1
	SNAI2	1.3	2.1	10.3	5.1
