# Supplementary material for: Leptin produced by obese adipose stromal/stem cells enhances proliferation and metastasis of estrogen receptor positive breast cancers
Source: Breast Cancer Res. 2015 Aug 19;17(1):112. doi: 10.1186/s13058-015-0622-z (PMC4541745; doi:10.1186/s13058-015-0622-z)
Supplement: Additional file 5: — mRNA expression of T47D cells after exposure to leptin knockdown adipose stromal/stem cells (ASCs). Data are shown as fold change relative to respective breast cancer cell line without previous co-culture with ASCs. *P <0.05; # P <0.01; ¥ P <0.001. lep leptin, ctrl control, lnASCs, adipose stromal/stem cells isolated from lean women, obASCs adipose stromal/stem cells isolated from obese women, EMT epithelial-to-mesenchymal transition (PDF 50 kb) [file 13058_2015_622_MOESM5_ESM.pdf]

Additional file 5

		T47D cells after co-culture with ASCs			
Function	*Gene Name*	ctrl shRNA *ln*ASCs	lep shRNA *ln*ASCs	ctrl shRNA *ob*ASCs	lep shRNA *ob*ASCs
Cell Cycle and Apoptosis	*CDKN2A*	2.8	2.7	1.5	1.9
	GSTP1	1.7	1.4	15.6*	30.4*
	SFRP1	4.0	0.6	0.7	0.7
Angiogenesis	*PLAU*	0.4	0.3	0.5	0.4
	THBS1	0.3	0.3	0.5	0.3
	CSF	0.3	0.3	0.5	0.3
EMT and Metastasis	*SERPINE1*	15.8*	15.3*	222.0[#]	17.6*
	MMP2	3.8	2.9	2193.0[¥]	2.6
	IL-6	2.2	2.2	14.0[¥]	9.0*
	TWIST1	5.6	4.2	19.6*	2.3
	PTGS2	0.0	0.1	0.0	0.0
	SNAI2	0.3	0.6	0.4	0.3
