# Supplementary material for: Leptin produced by obese adipose stromal/stem cells enhances proliferation and metastasis of estrogen receptor positive breast cancers
Source: Breast Cancer Res. 2015 Aug 19;17(1):112. doi: 10.1186/s13058-015-0622-z (PMC4541745; doi:10.1186/s13058-015-0622-z)
Supplement: Additional file 6: — mRNA expression of xenografts formed with MCF7 cells and leptin knockdown adipose stromal/stem cells (ASCs). Data are shown as fold change relative to respective breast cancer cell line without previous co-culture with ASCs. *P <0.05; # P <0.05; ¥ P <0.05. lep leptin, ctrl control, lnASCs, adipose stromal/stem cells isolated from lean women, obASCs adipose stromal/stem cells isolated from obese women, EMT epithelial-to-mesenchymal transition (PDF 49 kb) [file 13058_2015_622_MOESM6_ESM.pdf]

		Xenografts of MCF7 cells co-injected with	
Function	*Gene Name*	ctrl shRNA *ob*ASCs	lep shRNA *ob*ASCs
Cell Cycle and Apoptosis	*CDKN2A*	1.7	1.6
	GSTP1	1.2	1.4
	SFRP1	0.8	1.3
Angiogenesis	*PLAU*	2.0	0.8
	THBS1	3.0	3.0
	CSF	10.4[¥]	3.4[#]
EMT and Metastasis	*SERPINE1*	5.6[¥]	2.8[#]
	MMP2	2.8[¥]	1.4[#]
	IL-6	3.3	3.5
	TWIST1	0.3	0.6
	PTGS2	4.5	3.2
	SNAI2	13.8[#]	9.0*
